# Influence of rheumatoid factor levels and TNF inhibitor structure on secondary nonresponse in rheumatoid arthritis patients

**DOI:** 10.3389/fmed.2024.1461396

**Published:** 2024-09-04

**Authors:** Chamaida Plasencia-Rodríguez, Ana Martínez-Feito, Marta Novella-Navarro, Rebeca Pérez De Diego, Gema Bonilla, Johanna Elin Gehin, Alejandro Villalba-Yllán, Laura Nuño, Dora Pascual-Salcedo, Pilar Nozal, Mariana Díaz Almirón, Alejandro Balsa

**Affiliations:** ^1^Rheumatology Department, La Paz University Hospital, Madrid, Spain; ^2^Immuno-Rheumatology Research Group, Institute for Health Research (IdiPAZ), Madrid, Spain; ^3^Immunology Unit, La Paz University Hospital, Madrid, Spain; ^4^Laboratory of Immunogenetics of Human Diseases, IdiPAZ Institute for Health Research, La Paz University Hospital, Madrid, Spain; ^5^Innate Immunity Group, IdiPAZ Institute for Health Research, La Paz University Hospital, Madrid, Spain; ^6^Interdepartmental Group of Immunodeficiencies, Madrid, Spain; ^7^Department of Medical Biochemistry, Oslo University Hospital, Oslo, Norway; ^8^Center for Biomedical Network Research on Rare Diseases (CIBERER U754), Madrid, Spain; ^9^Biostatistics Unit, Institute for Health Research (IdiPAZ), Madrid, Spain

**Keywords:** rheumatoid arthritis, rheumatoid factor, TNF inhibitors, infliximab, adalimumab, certolizumab pegol, monoclonal antibodies

## Abstract

**Background:**

The EXXELERATE study revealed poorer clinical outcomes in patients treated with adalimumab (ADL) and baseline rheumatoid factor (RF) above 203 IU/mL. However, responses were similar in patients treated with certolizumab pegol (CZP) regardless of RF levels.

**Objectives:**

This study investigated the impact of RF levels >203 IU/mL on TNF inhibitors (TNFi) serum levels and the association with secondary nonresponse in RA patients treated with TNFi.

**Methods:**

We performed an observational ambispective study with RA patients treated with infliximab (IFX), ADL, or CZP. Patients were stratified according to baseline RF levels: ≤ or >203 IU/mL. After 6 months, serum drug levels and antidrug antibodies were measured, and reasons for discontinuation were collected.

**Results:**

We included 170 RA patients: 90 (53%) received IFX, 48 (28%) ADL, and 32 (19%) CZP. While CZP serum levels did not differ between RF groups at 6 months (*p* = 0.6), RF levels >203 IU/mL were linked to lower serum drug levels in patients treated with IFX (*p* = 0.09) or ADL (*p* = 0.02). Secondary nonresponse was 3.6 times higher in patients with high versus low RF levels in patients under IFX or ADL. However, the reasons for withdrawal were not affected by RF levels in patients treated with CZP.

**Conclusion:**

Baseline RF above 203 IU/mL is associated with lower serum drug levels and an increased risk of discontinuation due to secondary nonresponse in patients treated with IFX or ADL. In contrast, drug levels and clinical outcomes are not significantly impacted by baseline RF levels in patients under CZP.

## Introduction

Rheumatoid arthritis (RA) is a destructive systemic inflammatory joint disease that can cause chronic disability (1). It is characterized by the presence of autoantibodies such as rheumatoid factor (RF) and anti-citrullinated peptide antibodies (ACPA) (2), which are associated with increased severity of the disease (3, 4). Despite early treatment, RF and ACPA have been associated with greater structural progression in early RA patients (5).

According to the recommendations of the European League Against Rheumatism (EULAR) (6), biological or targeted synthetic disease-modifying antirheumatic drugs (b/tsDMARD) are recommended in RA patients whose therapy with methotrexate (MTX) fails (6). Tumor necrosis factor-alpha inhibitors (TNFi) are the most widely used biologics as a first-line option after failure of MTX. Although all TNFi types have the same mechanism of action, they have relevant structural differences (7): adalimumab (ADL), infliximab (IFX), and golimumab are monoclonal antibodies (MAB); etanercept is a fusion protein; and certolizumab pegol (CZP) is a pegylated Fab fragment (PEG) (7).

RF are antibodies with various isotypes and affinities that target the Fc portion of immunoglobulin G (IgG). They were first described in the first half of the 20th century (8). These antibodies have been linked to greater disease activity and are considered a risk factor for disease progression (5, 9–11). Moreover, high RF values are thought to predict discontinuation of TNFi due to inefficacy (12, 13). One of the most plausible hypotheses to explain these findings is that when RF binds the Fc region of TNFi, the resulting immune complexes are subsequently cleared from the bloodstream, leading to lower levels of circulating drug and reduced clinical efficacy (14, 15).

A *post hoc* analysis of the EXXELERATE study revealed poor clinical outcomes in patients treated with ADL whose baseline RF was above 203 IU/mL (16). However, responses to therapy were similar among patients treated with CZP, regardless of the baseline RF levels (16). Two recent real-world studies compared the influence of baseline RF levels on clinical outcomes of patients treated with CZP and those who received other types of TNFi (MAB and/or fusion protein) (17, 18). In the first one, a multicenter study carried out in a Spanish cohort, RF levels above 203 IU/mL were associated with a longer retention rate for CZP than for MAB (17). In the second study, serum drug levels after 6 months of starting the TNFi were independent of baseline RF levels in patients treated with CZP, although patients with high RF levels at baseline treated with MAB (IFX or ADL) had lower serum drug levels at 6 months than RF-negative patients (18). In addition, secondary nonresponse was more frequent in patients with high RF levels treated with MAB (18). In the same study, RF levels were divided into quartiles, and the RF cut-off point (203 IU/mL) previously described in the EXXELARATE study was not explored. Since these findings need to be validated in real-world cohorts, we aimed to investigate whether RF >203 IU/mL is associated with faster clearance of serum drug levels and an increased rate of withdrawal due to secondary nonresponse.

## Patients and methods

### Study design

We performed a real-world study with an observational ambispective design at the Complex Therapies Unit of the Rheumatology Department, La Paz University Hospital, Madrid, Spain. The study population comprised patients with RA who initiated biologic treatment with IFX, ADL, or CZP between 1999 and 2019. All patients enrolled fulfilled the American College of Rheumatology (ACR)/EULAR 2010 classification criteria for RA (19), were aged ≥18 years, had moderate or high disease activity (DAS28 >3.2), and satisfied the criteria of the Spanish Society of Rheumatology recommendations concerning the use of biological therapies in RA. Patients with concomitant inflammatory immune-mediated diseases other than RA, without serum samples at baseline and without available information about the reason of discontinuation were excluded. Additionally, to avoid a high number of missing data, it was required that more than 90% of the included patients had available clinical activity data and serum samples at 6 months after starting the TNFi. All participating patients gave their informed consent.

### Methods

The variables collected at baseline included demographic and clinical variables [age, sex, BMI, smoking status, duration of disease before initiation of bDMARDs, baseline 28-joint Disease Activity Score with erythrocyte sedimentation rate (DAS28-ESR), RF, ACPA, C-reactive protein (CRP)], in addition to concomitant and preceding treatment. Drug discontinuation was registered until December 2022 and classified according to reasons for withdrawal: primary nonresponse, secondary nonresponse, adverse events (AEs), and other.

RF and ACPA levels were measured at baseline using nephelometry (Siemens^®^), and ACPA was assessed using a commercial ELISA kit CCPImmunoscan (Svar Life Science^®^, Malmo Sweden). Serum drug levels of TNFi (IFX, ADL) and antidrug antibodies (ADAb) at 6 months were measured via commercial ELISA kits (Promonitor, Progenika Biopharma, Grifols^®^). CZP levels and anti-CZP antibodies were assessed using in-house, fluorometric assays automated on the AutoDELFIA immunoassay platform (PerkinElmer, Waltham, MA, United States) (20). Trough samples for analysis of serum drug and ADAb levels were collected before the following injection at baseline and at 6 months. During the first 6 months of follow-up, all TNFi were administered at standard doses for RA according to the technical information sheet (IFX at 3 mg/kg/iv at weeks 0, 2, and 6 and then every 8 weeks thereafter; ADL 40 mg sc every 2 weeks; CZP 400 mg sc at weeks 0, 2, and 4 and then 200 mg every 2 weeks thereafter). According to previous evidence from the EXXELARATE study, baseline RF was divided into low levels of RF (≤203 IU/mL) and high levels (>203 IU/mL) (16).

### Statistical analysis

Patients were divided into 2 groups for comparison between drugs, according to the biologic structure: the MAB group (IFX and ADL) and the PEG group (CZP). Continuous variables were expressed as the median and interquartile range (IQR) or mean and standard deviation (SD), whereas categorical variables were expressed as absolute and relative frequencies. The differences based on the treatment groups were evaluated using a 1-way ANOVA. *Post hoc* analysis (Tukey test) was performed when more than 2 groups were compared in the ANOVA test. The Mann–Whitney test was used to assess the relationship between RF values and serum levels of TNFi and between RF values and DAS28 at baseline. In patients with IFX and ADL, we used the chi-square test to examine differences in the percentage of ADAb-positive and negative patients. A Kaplan–Meier analysis was performed to evaluate drug survival and a competitive risk study adjusted for age, sex, MTX use, and baseline DAS28 was performed to analyze the risk of discontinuing the drug. Statistical significance was set at *p* ≤ 0.05. All statistical procedures were performed using IBM SPSS Statistics for Windows, Version 24.0 (IBM Corp., Armonk, NY, United States). Graphs were plotted using GraphPad Prism 6.0 software (GraphPad Prism Inc., La Jolla, CA, United States). R software version 4.3.1 (Vienna, Austria).

## Results

### Baseline clinical and demographic characteristics

A total of 415 patients with RA received treatment with IFX (*n* = 159), ADL (*n* = 148), and CZP (*n* = 32) between 1999–2019. Patients who did not have reported the cause of discontinuation, lacked serum samples at baseline and those with other associated immune-mediated diseases were excluded. Then, 245 patients were excluded and data from 170 patients were evaluated: 138 (81%) received MAB (90 with IFX and 48 with ADL) and 32 (19%) received PEG (CZP). More than 90% of the included patients had available serum samples at 6 months after starting the TNFi (*n* = 161) and clinical activity data at baseline and 6 months after starting TNFi treatment (*n* = 164). All patients had active disease at initiation of treatment (DAS28: 5.06 ± 1.35) (Table 1). Patients receiving PEG were numerically but not statistically significant older than those receiving MAB (*p* = 0.070). MTX use was numerically more frequent in the MAB group than in the PEG group (70% in MAB vs. 53% in PEG, *p* = 0.067). Although other DMARDs such as leflunomide, sulfasalazine, and hydroxychloroquine were used more frequently in the group of patients treated with PEG (18% in the MAB group vs. 47% in the PEG group, *p* = 0.001), none of the patients in the PEG group were on monotherapy. Twenty-six (15%) patients had previously received other biological treatment (22 were treated with TNFi, 1 with abatacept, 2 with tocilizumab, and 1 with a JAK inhibitor).

No significant differences were observed between baseline RF levels in naïve versus non-naïve patients treated with MAB and PEG [MAB, 102.5 (16.3–326.3) IU/mL in naïve vs. 55.4 (0–238.3) IU/mL in non-naïve patients, *p* = 0.309; PEG, 66.1 (17.8–346.8) IU/mL in naïve vs. 11.7 (0–102.8) IU/mL in non-naïve patients, *p* = 0.107].

### Disease activity (DAS28) stratified by RF levels at baseline

No significant differences in disease activity by DAS28 were detected in patients treated with MAB versus PEG (5.08 ± 1.36 in MAB vs. 4.98 ± 1.33 in PEG, *p* = 0.695). However, patients with a high-level of RF treated with PEG had more marked disease activity than those with a low level (PEG-DAS28, 5.95 ± 1.35 with HL with vs., 4.53 ± 1.09 with LL; *p* < 0.004). No significant differences were observed in DAS28 between baseline high and low levels of RF, respectively, in patients treated with MAB (MAB-DAS28, 4.99 ± 1.29 with HL vs. 5.27 ± 1.48 with LL, *p* < 0.282).

**Table 1:** Baseline demographic and clinical characteristics of patients treated with monoclonal antibodies (MAB) and pegylated (PEG) TNFi.

	Total *n* = 170	MAB *n* = 138	PEG *n* = 32	*p*
Sociodemographic characteristics				
Female sex, *n* (%)	141 (83%)	115 (83%)	26 (81%)	0.778
Age (years), median (IQR)	56.0 (45.8–66.1)	55.0 (44.8–65.0)	59.7 (47.7–70.1)	0.070
BMI, median (IQR)	24.6 (21.8–29.1)	24.3 (21.6–28.7)	24.7 (22.6–30.5)	0.561
Smoking status	*n* = 164	*n* = 136	*n* = 28	0.099
Current	27 (17%)	22 (16%)	5 (18%)	
Previous	40 (24%)	29 (21%)	11 (39%)	
Nonsmoker	97 (59%)	85 (63%)	12 (43%)	
Disease duration, (years)	8.5 (4.3–14.3)	8.5 (4.2–14.4)	8.9 (4.4–11.4)	0.921
Serological characteristics				
CRP mg/L, Median (IQR)	7.5 (3.0–21.4)	7.8 (3.0–22.8)	12.8 (2.4–17.6)	0.734
RF status (*n*, %)	128 (75%)	103 (75%)	25 (78%)	0.680
*RF levels (n, %)*				
Low: <203 IU/mL	117 (69%)	95 (69%)	22 (69%)	0.992
High: ≥203 IU/mL	53 (31%)	43 (31%)	10 (31.3%)	
ACPA status, (*n*/*N*, %)	135/168 (80%)	108/136 (79%)	27/32 (84%)	0.525
Clinical characteristics				
DAS28, mean ± SD	5.06 ± 1.35	5.08 ± 1.36	4.97 ± 1.33	0.480
Treatment characteristics				
Previous bDMARD use, *n* (%)	26 (15%)	20 (15%)	6 (19%)	0.547
Monotherapy, *n* (%)	16 (10%)	16 (12%)	0 (0%)	**0.042**
MTX use, *n* (%)	113 (67%)	96 (70%)	17 (53%)	0.067
Other DMARDs use, *n* (%)	40 (23%)	25 (18%)	15 (47%)	**0.001**
Prednisone use, *n* (%)	85 (50%)	69 (50%)	16 (50%)	0.970

RF, rheumatoid factor; ACPA, anti-citrullinated peptide antibodies; BMI, body mass index; DAS28, 28-joint Disease Activity Score 28; CRP, C-reactive protein; bDMARD, biologic disease-modifying drug; MTX, methotrexate.

### Baseline RF levels and serum levels of TNFi at 6 months

At 6 months, lower serum drug levels were reported in patients with high-level RF for both IFX (*p* = 0.09) and ADL (*p* = 0.02) (see Table 2). Nevertheless, CZP levels were comparable in patients with both low-and high-level RF (*p* = 0.6, see Table 2).

**Table 2:** Serum drug levels (ng/mL) at 6 months based on rheumatoid factor (RF) levels at baseline (*n* = 161 patients).

	Serum drug levels ng/mL [median (IQR)] at 6 months after starting TNFi^a^		*p*
	Baseline RF levels ≤203 IU/mL	Baseline RF levels >203 IU/mL	
Infliximab *n* = 85	376 [0–2,076]	0 [0–1,057]	0.09
Adalimumab *n* = 44	4,570 [1,104–7,572]	751 [53–2,401]	**0.02**
Certolizumab *n* = 32	33,000 [20,000–46,000]	32,000 [13,000–39,000]	0.6

TNFi, tumor necrosis factor alpha inhibitor; RF, rheumatoid factor. IFX, infliximab; ADL, adalimumab; CZP, certolizumab pegol; RF, rheumatoid factor.

a No serum sample was available to measure drug levels in 7 patients.

A subanalysis including only bDMARD-naïve patients (*n* = 144) revealed similar findings (see Supplementary Table S1).

### Immunogenicity of TNFi drugs at 6 months

Since there is an association between drug levels and immunogenicity, the development of ADAb was examined. At 6 months, ADAb were observed in 29 patients treated with MAB (26 with IFX and 3 with ADL) and in 1 patient with PEG. In the MAB group, more ADAb-positive patients were found in the group with a high level of RF at baseline (41% vs. 16%, *p* = 0.002).

### Association between discontinuation of therapy and baseline RF levels

Overall, 128 (75.3%) patients discontinued TNFi for different reasons: 24 (18.8%) owing to primary nonresponse, 66 (51.5%) owing to secondary nonresponse, 8 (6.3%) owing to AEs, and 30 (23.4%) owing to other causes (neoplasms, change of address, infections, surgeries, and pregnancy). The dropout percentage was slightly higher in patients treated with MAB that in those who received PEG [78% (108/138) vs. 63% (20/32), *p* = 0.063].

When comparing RF levels with the reasons for discontinuation, we observed that more patients with a high level of RF dropped out owing to secondary nonresponse than patients with low levels [74% (32/43) vs. 40% (34/85), *p* = 0.003]. This effect was observed in 79% of patients treated with MAB and in 40% of those treated with PEG, with the difference being statistically significant in the MAB group (*p* = 0.001) (see Supplementary Table S2).

### Comparison of drug survival between patients treated with MAB vs. PEG

Drug survival was shorter in patients with a high level of RF at baseline treated with MAB (3.9 ± 0.6 years vs. 6.5 ± 0.7 years, *p* = 0.037) than in the basal low RF level group. However, no significant differences in drug survival between RF groups were found with PEG (4.0 ± 0.6 years vs. 5.6 ± 1.5 years, *p* = 0.689).

**Figure 1:**
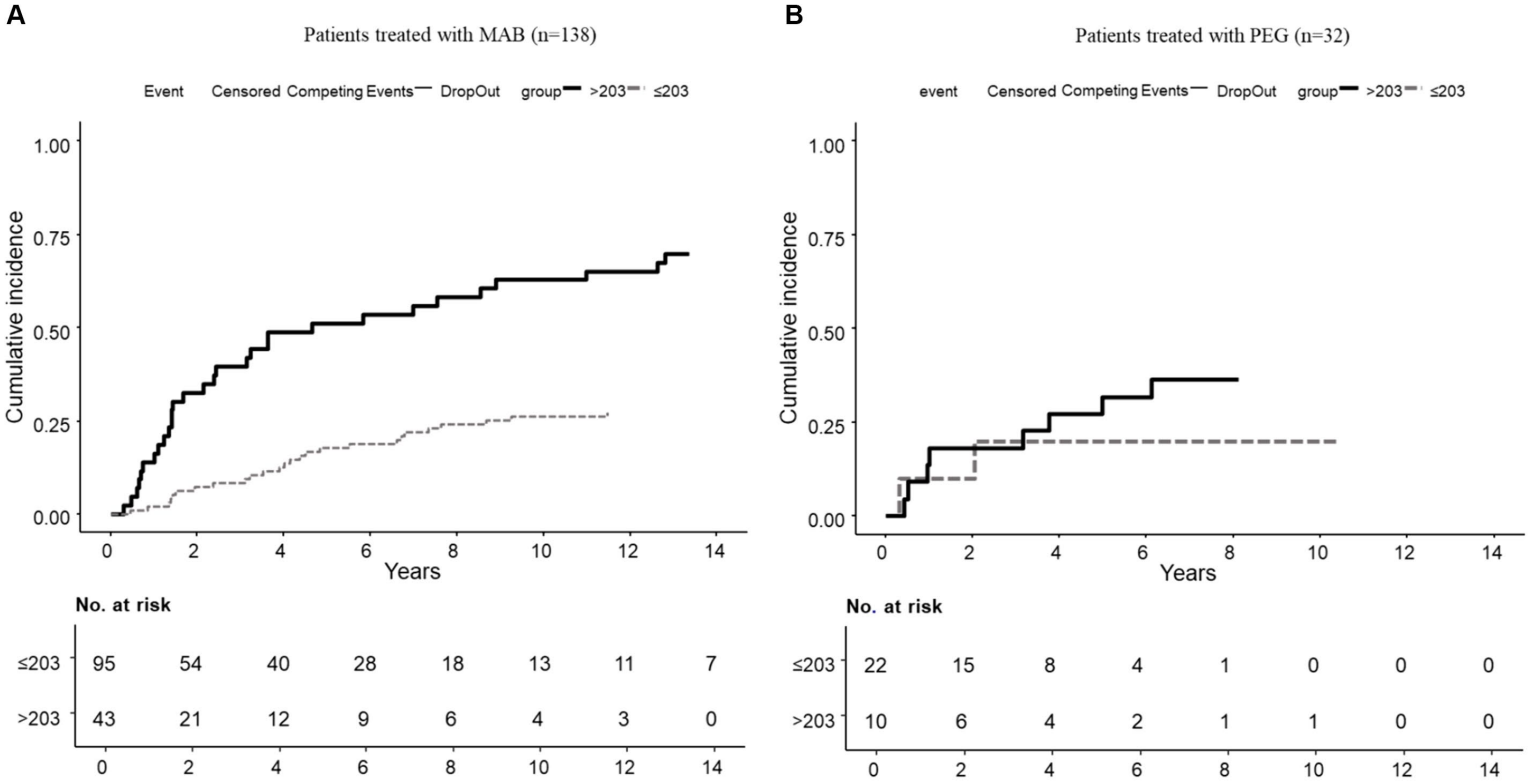
Cumulative incidence of dropout due to secondary nonresponse according to baseline RF levels. **(A)** In the MAB group (*n* = 138). **(B)** In the PEG group (*n* = 32).

The risk of dropout due to secondary nonresponse, according to a competitive risk study adjusted for age, sex, methotrexate, and baseline DAS28 increased 3.6 times in patients with RA treated with MAB and RF ≥203 IU/mL (*p* = 0.001) (see Figure 1A and Table 3). Nonetheless, no significant risk of dropout due to secondary nonresponse was found in patients with RA treated with PEG and RF ≥203 IU/mL (*p* = 0.445) (see Figure 1B and Table 3).

**Table 3:** Risk factors associated with discontinuation due to secondary nonresponse among patients treated with monoclonal antibodies (MAB) and pegylated antibody (PEG).

	HR	95% CI
Monoclonal antibodies (IFX and ADL) *n* = 138		
Baseline RF >203 IU/mL	**3.63**	**2.15–6.14**
No MTX use	0.73	0.42–1.30
Baseline DAS28	1.02	0.86–1.20
Age	1.01	0.99–1.02
Female sex	1.41	0.69–2.87
Pegylated antibody (CZP) *n* = 32		
Baseline RF >203 IU/mL	0.46	0.06–3.32
No MTX use	3.41	0.56–20.78
Baseline DAS28	1.35	0.59–3.06
Age	0.94	0.89–1.01
Female sex	3.42	0.72–16.22

HR, hazard ratio; IFX, infliximab; ADL, adalimumab; MTX, methotrexate; DAS28, 28-joint Disease Activity Score; CZP, certolizumab pegol.

## Discussion

This is the first real-world study to confirm that high baseline RF levels [>203 IU/mL (cut-off point proposed in the EXXELARATE study)] (16) are associated with secondary nonresponse in patients treated with MAB but not those treated with PEG. We observed that in patients with baseline RF levels greater than 203 IU/mL, MAB serum levels were lower and the discontinuation rate was higher owing to secondary nonresponse than in patients with lower RF titers. This effect was not observed in patients treated with PEG.

The advent of b/tsDMARDs represented a change in the clinical course and prognosis of patients with RA (21). Despite the large therapeutic arsenal, TNFi remain the biologics of choice, probably owing to experience of use, efficacy, and cost-effectiveness. However, around 30–40% of patients persist without achieving the therapeutic objectives of remission or low activity (22). On the other hand, available TNFi vary in terms of their pharmacological properties, with potentially different effects on clinical efficacy (23). These differential characteristics include pharmacokinetic aspects (half-life, clearance, or volume of distribution) and metabolism (23). Once MAB bind to their target, the resulting immune complexes can be recognized by the Fc receptor (FcR) and initiate signal transduction toward processes such as phagocytosis, antibody-dependent cell-mediated cytotoxicity, and complement-dependent cytotoxicity (23). Given the absence of the IgG1Fc domain, CZP does not elicit an antibody-dependent cell-mediated cytotoxicity response (24). Another aspect to highlight is that since MAB are bivalent and PEG are monovalent, the size of the immune complexes formed when binding to the target differs, with the result that clearance may be affected (25).

The formation and clearance of immunoclomplexes of TNFi drugs once they bind to their target is an exciting topic that could help us to better understand their clinical and paradoxical effects. Mechanistic insights into the factors contributing to differences in TNF clearance during treatment with TNFi were recently reported (26). In a cohort of patients with inflammatory arthritis or inflammatory bowel disease treated with TNFi, patients treated with CZP had the lowest concentrations of free TNF measured using a drug-tolerant ELISA (26). In addition, total TNF concentrations (free TNF and immunocomplexes TNF + TNFi) were higher in all patients with high drug levels throughout the first year of treatment, especially in those treated with CZP. This finding reflects the pharmacokinetic differences that confer the structure of TNFi (26). Another very relevant issue evaluated in this work is FcR binding *in vitro* (26). The authors observed how the TNF immune complexes formed with classic MAB are more likely to bind to the FcR than the TNF immune complexes formed with TNFi lacking the FAB portion (etanercept) (26). These assays confirm that binding between FcR and CZP is absent (26).

The previously discussed evidence leads us to ask whether pharmacological measures can optimize the pharmacokinetic properties of these treatments. Indeed, several mechanisms can be implemented to improve the pharmacokinetics of TNFi (25), including the fact that pegylation provides greater solubility, enhances resistance to proteolysis, increases bioavailability, and ensures better access to and permanence at the drug’s site of action (27, 28). Pegylation also reduces excretion through the kidneys and possibly immunogenicity, preventing agglomeration of the drug’s molecules and subsequent dysfunction and resulting in an increased half-life (27–29). CZP is the only PEG-TNFi, and it lacks the Fc fragment that interacts with the pre-existing RF. Both characteristics could explain part of the beneficial clinical effect that we observed among patients with high levels of RF (30).

The influence of RF on drug kinetics is a pivotal aspect of our discussion. Previous studies have posited that RF forms immune complexes with TNFi, leading to accelerated clearance from the bloodstream and, consequently, diminished drug efficacy (31, 32). Our results align with this hypothesis, highlighting the significant impact of high RF levels on serum drug concentrations and clinical outcomes. This observation is critical for clinicians considering TNFi, especially when choosing between MAB and PEG for patients with elevated RF. The use of RF as a diagnostic tool in patients with RA is clear, although its role in the monitoring of response to therapy is more limited (8). Published data on the potential role of RFs in predicting responses to TNFi are conflicting: some studies report that positive RF values before therapy are insufficient to predict a response (33–36), whereas others report that they predict a negative response (31, 37). In particular, high pretreatment levels of IgA RF are associated with poor clinical response (38). This controversy reflects the heterogeneity of published studies and the need to delve deeper into the underlying mechanisms to better understand the clinical consequences.

Several studies have evaluated the association between the effect of high RF levels on drug survival and response. Takeuchi et al. (39) provided valuable insights in this area, demonstrating that baseline RF and ACPA titers are associated with TNF levels and subsequent clinical responses to infliximab. A *post hoc* analysis from the EXXELERATE study revealed poorer clinical outcomes measured by DAS28-CRP in patients treated with ADL and baseline RF above 203 IU/mL (16). However, the response to therapy was similar regardless of RF levels in patients treated with CZP (16). In a recent multicenter study, better retention rates were observed with PEG in patients with baseline RF levels above 203 IU/mL than with other TNFi (17). Nonetheless, the association between reasons for discontinuation and RF levels was not assessed in that multicenter study (17). In a previous study by our group, we found that patients with higher RF levels had faster drug clearance and a higher proportion of discontinuation due to secondary nonresponse than RF-negative patients (18). However, these results had not been validated with the cut-off above 203 IU/mL described in the EXXELARATE study (16). In the present work, baseline RF above 203 IU/mL is associated with greater clearance and poorer retention of MAB than of PEG. Interestingly, this cut-off is independently associated with an almost 4 times higher risk of secondary nonresponse in patients treated with MAB. These findings lead us to reflect on the need to measure RF levels before starting a TNFi in order to ensure the option that leads to the greatest long-term effectiveness.

Immunogenicity plays a decisive role in the response to biological therapy. Development of ADAb is an important pharmacodynamic factor and is closely related to the structure and composition of MAB, dosage, route of administration, and comedication (40). However, the clinical impact of detecting ADAb in patients treated with CZP is less clear. In a recent publication including 40 RA patients treated with CZP, high CZP concentrations were also recorded in most cases where ADAb were detected (41). In our cohort, the frequency of ADAb to IFX and ADL is consistent with the literature (42), and the frequency of anti-CZP antibodies in clinical trials of RA patients ranges from 5 to 8% (42–45); in the NOR-DMARD study, the incidence was 6% at 3 months (46). In our cohort of patients treated with MAB, we observed a higher proportion of ADAb positive patients in the group of RF >203 IU/mL. However, this involves a very limited number of patients, and the influence of ADAb in patients with high RF levels treated with TNFi remains unclear in the literature.

Our study is not without limitations. The retrospective design, the limited number of patients in some of the treatment groups and potential confounding factors, such as variations in adherence and the influence of concomitant medications may affect the generalizability of our findings. Therefore, to minimize the effect of these biases, confounding variables such as disease activity, age, sex, and concomitant treatment were adjusted for in the survival analysis. Another limitation is that a group of patients treated with etanercept was not included. This decision was based on the fact that this drug is a fusion protein and, therefore, less immunogenic than the other TNFi drugs. Additionally, the specific impact of RF isotypes and affinity on the kinetics of TNFi warrants further investigation.

In conclusion, our study supports the relevance of RF levels above 203 IU/mL for predicting clinical response and survival of TNFi therapies in patients with RA treated with TNFi in clinical settings. These findings advocate for a more nuanced approach to RA treatment, emphasizing the need for personalized therapeutic strategies based on individual biomarker profiles. Future research should aim to elucidate the mechanisms underlying the interaction between RF and TNFi, potentially paving the way for novel therapeutic approaches tailored to the immunological landscape of RA patients.

## Data Availability

The raw data supporting the conclusions of this article will be made available by the authors, without undue reservation.
