# Supplementary material for: Influence of rheumatoid factor levels and TNF inhibitor structure on secondary nonresponse in rheumatoid arthritis patients
Source: Front Med (Lausanne). 2024 Sep 4;11:1461396. doi: 10.3389/fmed.2024.1461396 (PMC11410080; doi:10.3389/fmed.2024.1461396)
Supplement: Supplementary file 1 [file Data_Sheet_1.PDF]

Supplementary material:

Supplementary Table 1. Serum drug levels (ng/ml) at 6 months based on rheumatoid factor levels at baseline only in bDMARD-naïve patients (n=144 patients)

	**Serum drug levels at 6 months of starting TNFi**		**p**
	**Baseline RF levels ≤200 IU/ml**	**Baseline RF levels >200 IU/ml**	
**Infliximab n=76**	851.6 [12.0-2354.1]	9.8 [0.0-1137.2]	0.053
**Adalimumab n=34**	4548.0 [1645.5-9550.5]	128 [13.8-4440.4]	0.071
**Certolizumab n=26**	39000.0 [19000.0-48000.0]	31000.0 [21500.0-41500.0]	0.426

bDMARD: biologic disease-modifying antirheumatic drug; TNFi: tumor necrosis factor alpha inhibitor; RF: rheumatoid factor.

Supplementary Table 2: Reasons for discontinuing TNFi treatment according to baseline RF levels.

	Reasons for discontinuation				p
	**Primary nonresponse**	**Secondary nonresponse**	**Adverse events**	**Other**	
	**Monoclonal antibody (IFX and ADL)**				
**Baseline RF ≤200 IU/ml n=70**	19 (27%)	26(37%)	6 (9%)	19 (27%)	**0.001**
**Baseline RF >200 IU/ml n=38**	3 (8%)	**30 (79%)**	1 (3%)	4 (10%)	
	**Pegylated antibody (CZP)**				
**Baseline RF ≤200 IU/ml n=15**	1 (7%)	8 (83%)	1 (0%)	5 (10%)	0.758
**Baseline RF >200 IU/ml n=5**	1 (20%)	2 (40%)	0 (0%)	2 (40%)	
